# Characterization of a Synaptic Vesicle Binding Motif on the Distal CaV2.2 Channel C-terminal

**DOI:** 10.3389/fncel.2016.00145

**Published:** 2016-06-09

**Authors:** Sabiha R. Gardezi, Arup R. Nath, Qi Li, Elise F. Stanley

**Affiliations:** Laboratory of Synaptic Transmission, Krembil Research Institute, TorontoON, Canada

**Keywords:** presynaptic, calcium channel, synaptic vesicle, tether, SV-PD, CaV2.2, transmitter release, C-terminal, binding motif

## Abstract

Neurotransmitter is released from synaptic vesicles (SVs) that are gated to fuse with the presynaptic membrane by calcium ions that enter through voltage-gated calcium channels (CaVs). There is compelling evidence that SVs associate closely with the CaVs but the molecular linking mechanisms remain poorly understood. Using a cell-free, synaptic vesicle-pull-down assay method (SV-PD) we have recently demonstrated that SVs can bind both to the intact CaV2.2 channel and also to a fusion protein comprising the distal third, C3 segment, of its long C-terminal. This site was localized to a 49 amino acid region just proximal to the C-terminal tip. To further restrict the SV binding site we generated five, 10 amino acid mimetic blocking peptides spanning this region. Of these, HQARRVPNGY effectively inhibited SV-PD and also inhibited SV recycling when cryoloaded into chick brain nerve terminals (synaptosomes). Further, SV-PD was markedly reduced using a C3 fusion protein that lacked the HQARRVPNGY sequence, C3HQless. We zeroed in on the SV binding motif within HQARRVPNGY by means of a palette of mutant blocking peptides. To our surprise, peptides that lacked the highly conserved VPNGY sequence still blocked SV-PD. However, substitution of the HQ and RR amino acids markedly reduced block. Of these, the RR pair was essential but not sufficient as the full block was not observed without H suggesting a CaV2.2 SV binding motif of HxxRR. Interestingly, CaV2.1, the other primary presynaptic calcium channel, exhibits a similar motif, RHxRR, that likely serves the same function. Bioinformatic analysis showed that variations of this binding motif, +(+) xRR (where + is a positively charged aa H or R), are conserved from lung-fish to man. Further studies will be necessary to identify the C terminal motif binding partner on the SV itself and to determine the role of this molecular interaction in synaptic transmission. We hypothesize that the distal C-terminal participates in the capture of the SVs from the cytoplasm, initiating their delivery to the active zone where additional tethering interactions secure the vesicle within range of the CaV single Ca^2+^ domains.

## Introduction

**FIGURE 1:**
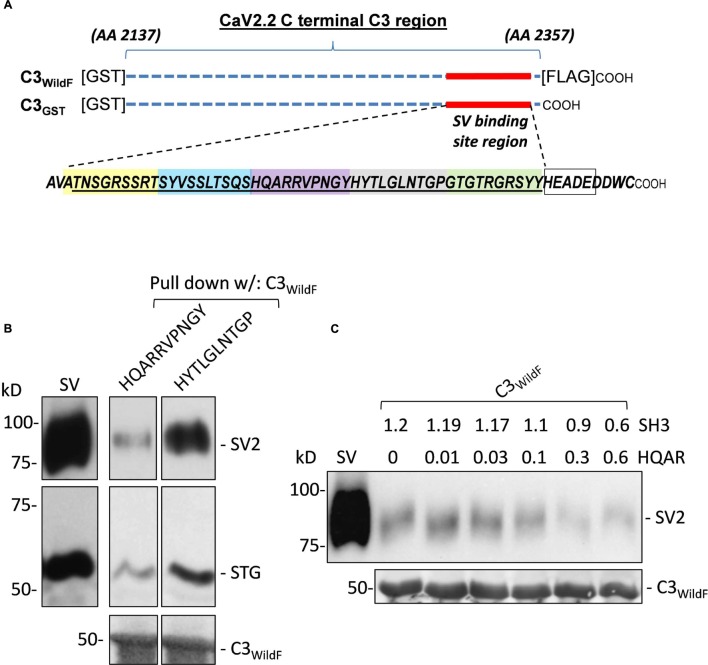
**(A)** Diagram of the full-length C3 fusion proteins used in this study with the synaptic vesicle (SV) binding site and the synthetic mimetic peptides. GST-tagged fusion proteins, C3_WildF_ and C3_GST_, comprising approximately the distal third of the CaV2.2 C-terminal, are indicated by the horizontal dashed lines. The two fusion proteins are identical except for a FLAG tag on the distal tip of the former. The 49 aa sequence of the SV binding region is indicated together with the 9 aa (HEADEDDWC) channel terminus. The colored boxes define the five mimetic blocking peptides synthesized to identify the SV binding motif. The location of the HEADE peptide sequence is also indicated within the HEADEDDWC region (empty box). **(B)** Preliminary experiment showing inhibition of SV-PD by a mimetic peptide. Sucrose gradient-purified SV samples in a detergent-free buffer were pre-incubated with test HQARRVPNGY or HYTLGLNTGP peptides (1.2 mM) prior to SV-PD with immobilized C3_WildF_ fusion protein. SV capture was assessed by Western blotting (WB) for the recovery of at least two integral SV proteins: SV2 and STG. SV-PD was considerably less with HQARRVPNGY in comparison to HYTLGLNTGP. Equal loading of the fusion protein was confirmed using an anti-GST tag antibody. **(C)** Optimization of peptide concentration for detection of SV-PD block. Based on preliminary experiments, HQARRVPNGY (HQAR) was used to test for a suitable peptide concentration for effective block. SVs were pre-incubated with HQARRVPNGY peptide with a control peptide, SH3 (which was used as the control in all experiments, unless indicated), added in reciprocal amounts to maintain a total buffer peptide concentration of 1.2 mM (*N* = 1). SV-PD was carried out as described and SV capture was assayed by blot for SV2 or STG (not shown). Note the decline in SV2 recovery, corresponding to the reduction in SV capture, at HQARRVPNGY concentrations greater than 0.03 mM.

Calcium ion entry through presynaptic voltage-sensitive CaV2.2 calcium channels is known to gate transmitter release by the fusion and discharge of transmitter from docked synaptic vesicles (SVs) (Stanley, 2016). The finding that SV fusion can be gated by a single CaV2.2 led to the prediction that the channel and SV are linked by at least one protein tether (Stanley, 1993) and a number of studies have explored this molecular interaction. Two main molecular tethering linkage mechanisms have been considered; first, a link involving integral surface membrane proteins, in particular the SNARE protein syntaxin 1 (Sheng et al., 1998; Seagar et al., 1999; Mochida et al., 2003) and a surface membrane-independent, cytoplasmic link from the channel directly to the SV (Kaeser et al., 2011; Wong et al., 2013, 2014). A novel, cell-free synaptic vesicle-pull down assay (SV-PD) demonstrated for the first time that SVs can bind to CaV2.2 by a direct, membrane-independent mechanism (Wong et al., 2013). This study also showed that intact SVs can be captured by a synthetic fusion protein comprising the distal third (C3) of the CaV2.2 channel C-terminal (Wong et al., 2013). A later study used truncated or PDZ ligand domain-modified C3 constructs and mimetic blocking peptides to identify a 49 amino acid residue (aa) SV binding region, 9 aa proximal to the C-terminal distal tip (Wong et al., 2014), as indicated in **Figure 1**.

In this study we used mimetic blocking peptides that span the SV binding region to search for specific SV attachment motifs. We then tested if these peptides would interfere with SV-PD by blocking the (unknown) SV binding site. In a complimentary study we also to tested if the peptides affected presynaptic SV recycling in functional isolated brain nerve terminals (synaptosomes, SSMs) using a ‘peptide cryoloading’ method combined with a styryl dye SV recycling assay (Nath et al., 2014). Finally, we used a combination of bioinformatics analysis comparing release site-associated CaV types in a range of species together with mutated peptide blockers to identify a putative SV binding motif.

## Materials and Methods

### Synaptic Vesicle Binding Assays

#### Synaptosome and Synaptic Vesicle Fractionation

The synaptosome and SV fractionation method has been described in detail (Wong et al., 2013). Briefly, E14–E17 chick brains (typically 100 per preparation) were homogenized and the SVs purified by differential and sucrose density gradient centrifugation. The SVs were maintained intact in detergent-free buffer for all experiments. Key buffers were: homogenization buffer (HB), 0.32 M sucrose, 10 mM HEPES, 2 mM EDTA, pH 7.4; and HEPES-lysis buffer, 50 mM HEPES, 2 mM EDTA, pH 7.4 (Wong et al., 2013, 2014).

#### Antibodies

Antibodies used in the present study are listed in **Table 1**.

**Table 1:** Antibodies.

Antibody	Target	Source (WB dilution)	WB dilution
FLAG (m)	FLAG tag	Sigma–Aldrich	1:4000
GST (m)	GST tag	Santa Cruz Biotechnology	1:4000
L4569 (p)	C-terminal of CaV2.2 long splice variant	Stanley lab (Khanna et al., 2006)	1:1000
RIM2 (RIM1/2; p)	RIM (RIM1 and 2; RIM1/2)^∗^	Synaptic Systems, GMBH	1:2000
SV2A (m)	SV2A (SV2)	Synaptic Systems, GMBH (clone 171G0)	1:1000
Synaptotagmin (m)	Synaptotagmin-1 (STG)	Abcam (clone ASV30)	1:1000
VAMP (p)	Vesicle Associated Membrane Protein 1,2,3 (VAMP)	Synaptic Systems, GMBH	1:2000
V-ATPase (p)	V-ATPase (V0) A1	Santa Cruz Biotechnology	1:1000


*^∗^RIM2 antibody has been characterized previously. See Wong and Stanley (2010).*

### Western Blot

Standard Western blotting (WB) method was carried out as described (Wong et al., 2013, 2014) except immunoblots were imaged using a ChemiDoc^TM^ XRS System (Bio-Rad).

### Protein Detection

Mini-PROTEAN TGX ‘Stain-Free’ Precast Gels (4–15%, Bio-Rad) were used to C3WildF and C3HQless fusion protein dilution analysis. The samples were separated using SDS-PAGE as previously described (Wong et al., 2013, 2014). The gels were than UV activated according to the manufacturer’s protocol using the ChemiDoc XRS. The fusion protein bands were visualized within 0.5 s after gel activation.

### Synaptic Vesicle Pull-Down Assay

The SV-PD assay has been described in detail (Wong et al., 2013, 2014). Briefly, purified SVs were incubated with immobilized C3 fusion proteins or GST control in a detergent free, SV-PD buffer (HB with 5 mM EGTA and free Ca^2+^ clamped to 10 nM, CaCl_2_ was calculated using MaxChelator). Prior to SV-PD, 40 μl of the SV suspension, containing the SV sample used for pull-down assays, was reserved for WB. SV-PD samples were washed four times with SV-PD buffer and solubilized in 4X Laemmli sample buffer with 5% β-mercaptoethanol for WB. Immunoblots were probed for integral SV membrane proteins as markers for vesicle capture. SV-PD was considered positive if the band intensity of two of the vesicle integral membrane marker proteins, generally SV2 and STG, were more intense than for control samples (Wong et al., 2013, 2014). The use of freshly prepared SVs and detergent-free buffers makes these both time-demanding experiments with some non-specific SV capture with control beads and fusion proteins (Wong et al., 2014). Criteria for acceptance for further analysis were stringent based on a rejection of blots with a significant level of non-specific binding to controls.

### CaV2.2 Distal C-terminal Region Mimetic Peptides

**FIGURE 2:**
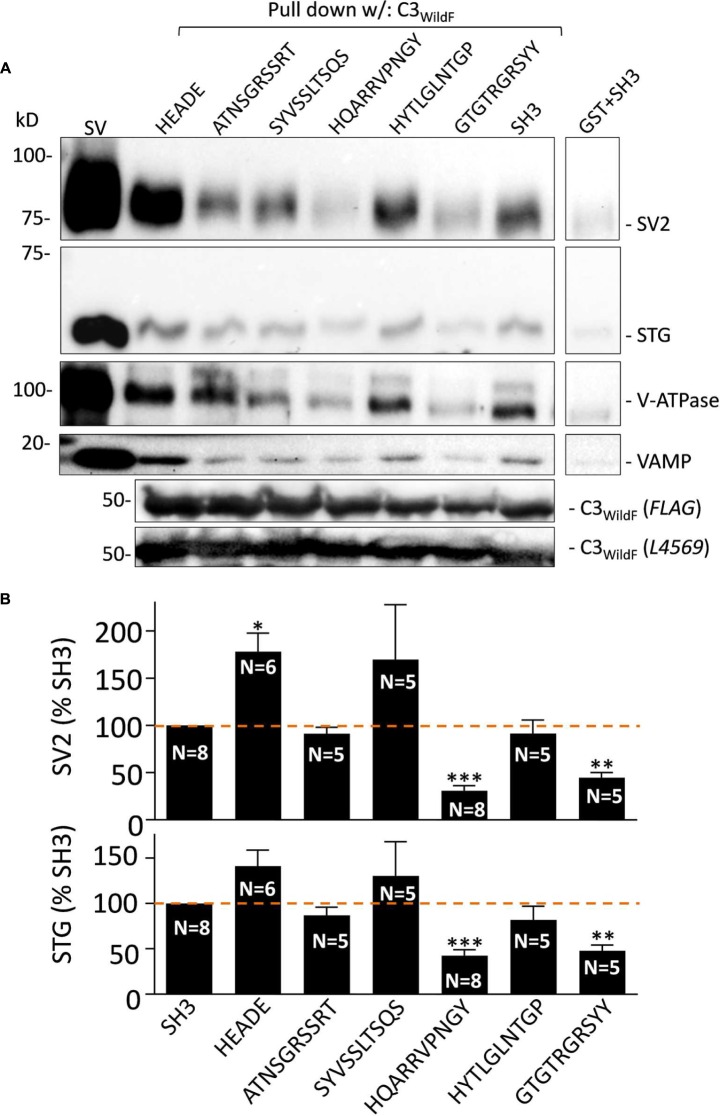
**(A)** Effect of the mimetic blocking peptides on SV-PD with C3_WildF_. Purified SVs were pre-treated with each of the five mimetic blocking peptides or HEADE and SH3 controls (0.6 mM) prior to SV-PD with C3_WildF_ fusion protein. SV-PD was assessed by the vesicle marker proteins, SV2 and STG. We also blotted for two additional integral proteins, V-ATPase and VAMP. Compared to the SH3 peptide control, integral SV protein band intensities were reduced by HQARRVPNGY and GTGTRGRSYY, increased by HEADE and minimally affected by ATNSGRSSRT, SYVSSLTSQS, or HYTLGLNTGP. Protein bands were faint with a purified GST control. C3_WildF_ fusion protein was identified by either anti-FLAG or anti-CaV2.2 (L4569) antibodies. **(B)** Quantification of mimetic peptide effect on SV-PD. SV2 and STG protein band intensities were normalized to the SH3 control peptide on the same immunoblot. Column graphs of SV2 (top) and STG (bottom) show percent mean ± SE of *N* separate experiments. HQARRVPNGY (*N* = 8) and GTGTRGRSYY (*N* = 5) peptides inhibited SV capture as indicated by both vesicle marker proteins (SV2, top; STG, bottom). SYVSSLTSQS shows a large variability between experiments due to a single outlier; if omitted the mean was 118 ± 35%. HEADE peptide resulted in a significant enhancement in the mean SV2 band intensity (SYVSSLTSQS *N* = 5; HEADE, *N* = 6). *t*-test, ^∗^*p* < 0.05; ^∗∗^*p* < 0.01, ^∗∗∗^*p* < 0.001.

**FIGURE 3:**
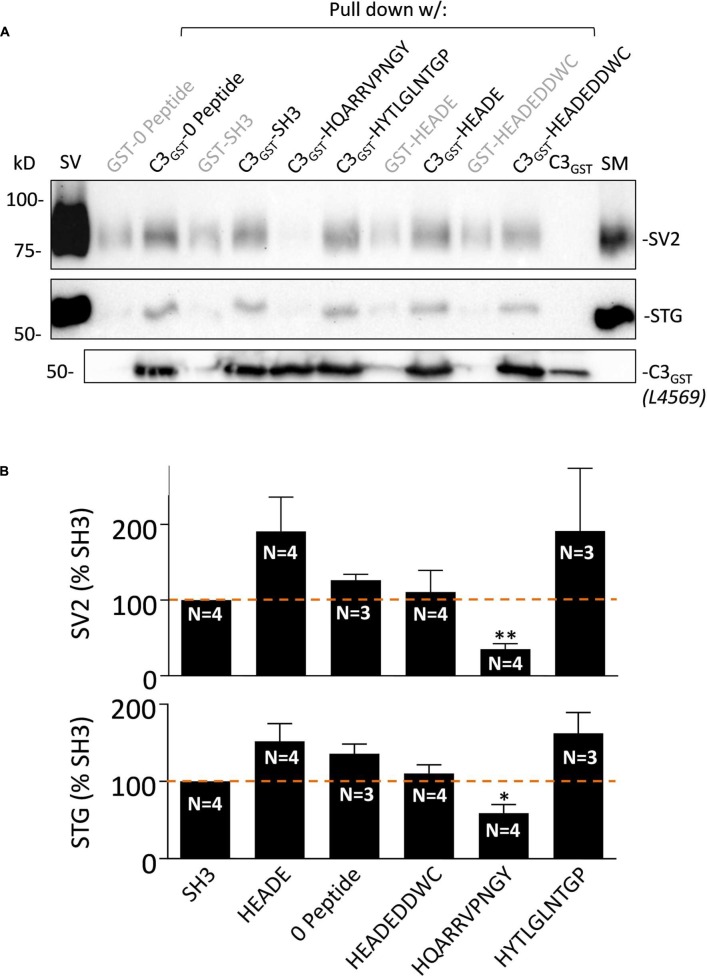
**HQARRVPNGY inhibits SV-PD with native C3 fusion protein.** **(A)** The effect of HQARRVPNGY on SV capture as in **Figure 2** was repeated using C3_GST_ probing for SV2 and STG (0.6 mM peptide). **(B)** The peptide inhibited SV-PD compared to SH3 peptide whereas three control peptides, a zero peptide control and also one other test peptide, HYTLGLNTGP, had no effect. Both SV2 and STG exhibited increased mean band intensities with HEADE but this did not reach statistical significance (*t*-test, *p* > 0.1) while HEADEDDWC was unchanged. ^∗^*p* < 0.05, ^∗∗^*p* < 0.01.

Control and putative blocking peptides were synthesized at the SPARC BioCentre (Toronto, ON). Control peptides for this study have been described (Wong et al., 2014): RQLPQTPL (SH3, aa 2210–2217), HEADEDDWC (aa 2349–2357), and HEADE (aa 2349–2353). Five peptides spanning the SV binding site region were: ATNSGRSSRT (aa 2299–2308), SYVSSLTSQS (aa 2309–2318), HQARRVPNGY (aa 2319–2328), HYTLGLNTGP (aa 2329–2338), and GTGTRGRSYY (aa 2339–2348; **Figure 1A**). Peptides were reconstituted in HB at 10 mM. SVs were incubated with the peptides for 2 h at 4°C prior to pull-down with the fusion proteins. To establish a suitable peptide concentration, we carried out SV-PD trials using C3_WildF_ in the presence of HQARRVPNGY, which was shown to block in preliminary experiments (**Figure 1B**). To avoid complications due to variations in total soluble peptide we included a control peptide, SH3, or HEADE, to maintain the total peptide concentration at 1.2 mM. Previous experiments demonstrated that neither of these inhibit SV-PD (Wong et al., 2013). In a preliminary experiment we found that 50% maximal inhibition of SV-PD was observed at ∼0.3 mM, as assessed by SV2 recovery, with both control peptides (SH3, **Figure 1C**; HEADE *Data not shown*) and we therefore used a 0.6 mM peptide for all blocking experiments, except as stated. During the course of these experiments we noted that one of the control peptides, HEADE, a sequence within the C-terminal distal to our primary area of interest, actually enhances SV-PD (**Figures 2** and **3**). This effect was not observed with the SH3 or HEADEDDWC – a C-terminal tip peptide that includes HEADE (**Figure 3**). Thus, while this finding is of interest in itself, the latter makes it less likely that enhancement in SV-PD observed with HEADE is of biological significance. Nonetheless, after this finding we stopped using HEADE as a control in the peptide-block experiments.

We also generated a series of blocking peptides to zero in on the C3 region SV binding motif. These peptides were: HQARRAPNGA, HQARRGPNGG, HQARRAGGGA, HQARR GAAAG, HQARRAAAAA, HQAAAVPNGY, HQAGGAGGGA, AAARRVPNGY, and GGARRAGGGA (see below).

### Immunoblot Quantification and Analysis

Immunoblots were probed for integral vesicle proteins, SV2, and STG, which were used as markers for SV capture in this study. Immunoblots were imaged with the ChemiDoc (Bio-Rad) with a broad range of exposure times. For each experiment, protein band intensities were quantified by densitometry using Image Lab (Bio-Rad) software from a common blot at a single exposure selected for clear bands without saturation. Background counts were subtracted using an automated routine and protein band intensities of test (*I*_x_) or control (*I*_c_) peptide treatments were measured. Percent integral protein capture, and hence %SV-PD, was calculated as: (*I*_x_/*I*_c_) × 100.

All experiments were pre-hoc testing treated samples to a defined control. Data are presented as mean +SE (%) and “*N*-value” represents the number of independent experiments. Statistical analysis was performed with GraphPad Prism 6.0. Each peptide treatment was tested using a one sample, two-tailed *t*-test based on the null hypothesis that mean SV-PD = 100% (that is equal to the control peptide), as described (Wong et al., 2013). Data was also tested with a *post hoc* ANOVA adjusted with a Bonferroni–Holms correction for multiple comparisons^1^. Values were considered significantly different if *p* < 0.05.

### Functional Assay of Synaptic Vesicle Recycling

#### Cryoloading

The method has been described (Nath et al., 2014). Briefly, SSMs were isolated as described above. SSMs were pelleted in SET buffer (0.32 M sucrose; 1 mM EDTA; 5 mM Tris) in preparation for cryoloading. 1.2 mM of the blinded mimetic peptide (or equivalent control) was added to the SSM mixture (50 μL total volume) with 20 μM of the 3 kD Dextran-FITC loading maker and was frozen slowly in a –80° freezer.

#### Styryl Dye Uptake

Vesicle turnover was assessed using a standard dye uptake method (Nath et al., 2014). Briefly, thawed SSMs were plated in Krebs-like physiological buffer (KPB: 143 mM NaCl, 4.7 mM KCl, 1.3 mM MgSO_4_, 1.2 mM CaCl_2_, 20 mM HEPES, 0.1 mM NaH_2_PO_4_, 10 mM glucose; pH 7.4) and depolarized for 2 min at 30°C using 40 mM K^+^ in the presence of 1 μM FM4-64 (Invitrogen). SSMs were washed with KPB supplemented with 1 mM Advasep-7 (Sigma–Aldrich) prior to mounting in DAKO mounting medium (DAKO) for fluorescence imaging.

#### Microscopy

Imaging was carried out on a Zeiss Axioplan2 with a 63×, 1.4 NA objective.

### Ethics Statement

Only chick embryos were used in this study. The University Health Network TG&W Animal Care Committee has granted a waiver to perform these experiments as the embryos used in this study were all in early stages of embryonic development and before day 21 and therefore committee review and approval was not necessary. However, the study was reviewed by a veterinarian and the euthanasia procedure was validated.

### Peptide Mimetic Blocker Experimental Design and Analysis

The experiment and data analysis were carried out blind. A laboratory member unconnected with the SV recycling experiment assigned an alphabet code to each test peptide and the code was broken on completion of analysis. The styryl dye uptake quantification method has been described (Nath et al., 2014). Briefly, a minimum of three images per treatment per experimental trial were quantified. Cryoloaded single SSMs were identified by positive stain for the loading marker (FITC-dextran) and were imaged by bright field to ensure isolation from un-loaded neighbors. Each FITC-stained SSM was scored as FM4-64 positive or negative by eye and the ratio of positive over total examined was used to calculate the percentage of SV recycling SSMs.

The fraction of dextran-positive control SSMs that showed styryl dye uptake was consistent between experimental trials and across treatments (no-load, 61.3 ± 3.2; HEADEDDWC 59.5 ± 1.6) and hence, normalization was not necessary. Student’s *t*-test was used to compare test peptide-treated to both the No-load and HEADEDDWC peptide controls.

## Results

### SV Binding Assay, Bait Proteins, and C-terminal Mimetic Blocking Peptides

#### The SV-PD Assay

We have described a cell-free assay, termed SV-PD, to test if SVs can be captured by the intact CaV2.2 channel. Briefly, fresh SVs are purified by differential centrifugation on discontinuous sucrose gradients for each experiment. The SVs are then incubated with an immobilized bait, in the present case the distal third of the channel C-terminal (C3 region), in a detergent-free buffer. Successful SV capture is assessed by Western blot for integral SV proteins (SV2, STG, VAMP, etc).

#### CaV2.2 distal C-terminal Fusion Proteins

Most of these experiments were carried out using the *N*-terminal-GST-tagged fusion protein, C3_wildF_ (**Figure 1A**), as the bait. C3_wildF_ fusion protein is identical to the chick C-terminal C3 region except for the addition of a FLAG tag to the C-terminal tip (Wong et al., 2014) and was used for most of this study. We also generated a new fusion protein, C3_GST_ (**Figure 1A**, see below) which is identical to the native C-terminal but differs from C3_wildF_ by the absence of the FLAG tag. Both fusion proteins were purified with high yield by our bacterial expression system and were robust for SV-PD.

#### Blocking Peptides

To further restrict the SV attachment site within the previously identified 49 aa region of the C3 terminal region we synthesized five abutting 10 aa mimetic blocking peptides (**Figure 1A**). Beginning from the N terminal end these were: ATNSGRSSRT, SYVSSLTSQS, HQARRVPNGY, HYTLGLNTGP, and GTGTRGRSYY. The peptide blockers were designed to be non-overlapping to permit their use in combinations while minimizing complication due to mutual binding interference.

#### Effect of Single Mimetic Peptides on SV-PD

##### A subset of the SV binding region mimetic peptides inhibited SV-PD

To identify which segment of the SV binding region plays a role in SV binding we carried out SV-PD experiments in the presence of each of the five peptides separately (**Figure 2A**). SV2 and STG bands were quantified by densitometry (**Figure 2B**) and normalized to a control SH3 peptide-treated band.

The results with SV2 and STG were comparable but the immunoblot signal-to-noise ratio was higher with the former. The blots showed that SV-PD was not significantly inhibited by ATNSGRSSRT, SYVSSLTSQS (which exhibited large variability), or HYTLGLNTGP. However, it was reduced significantly by HQARRVPNGY (*p* < 0.001) and by GTGTRGRSYY (*p* < 0.01; **Figure 2B**).

#### HQARRVPNGY Inhibits SV Capture with Native C3 Fusion Protein

The fusion protein used in the above studies, C3_WildF_ is identical to the native, long splice chick CaV2.2 aa sequence but with a FLAG tag on its terminus. To increase confidence in our results and to rule out the remote possibility that the FLAG tag introduced anomalous results we repeated the experiment with C3_GST_ which lacks this tag. This fusion protein also captured SVs, as assessed by SV-PD (**Figure 3A**) and, consistent with the findings with C3_WildF_, this was markedly inhibited by HQARRVPNGY but not by another binding region peptide, HYTLGLNTGP, as compared to no-peptide or SH3 (**Figure 3B**). Compared to the no-peptide or SH3 controls, the mean SV-PD was higher with HEADE and HYTLGLNTGP, but neither reached significance (*p* > 0.1 for both SV2 and STG). No significant effect was observed with the HEADEDDWC control peptide (*p* > 0.1).

#### SV Recycling Is Inhibited by Single Peptides

**FIGURE 4:**
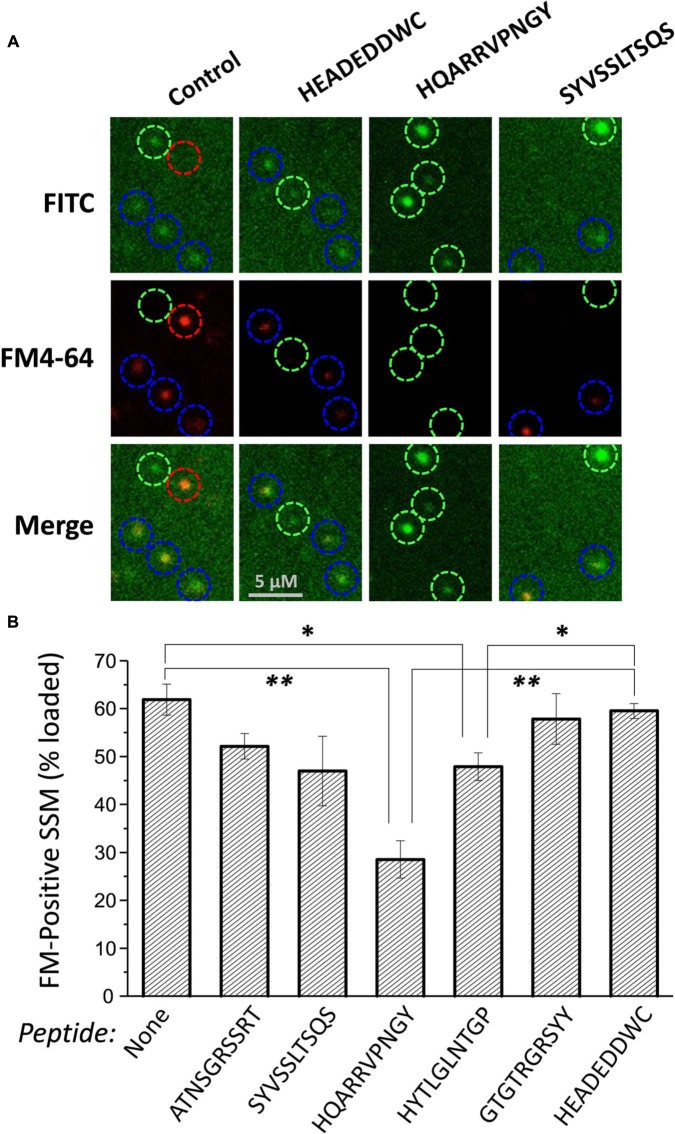
**Effect of mimetic peptides on SV recycling.** SSMs were cryoloaded in a blinded manner with the indicated peptides (1.2 mM) together with 3 kD Dextran-FITC (20 μM). The cryoloaded terminals were depolarized with 40 mM K^+^ in the presence of 1.2 mM Ca^2+^ to trigger exocytosis and uptake of FM4-64 (1 μM) by vesicle turnover. The ***FITC*** panels show SSMs that have been cryoloaded with the loading marker and the ***FM4-64*** panels show SSMs that have taken up styryl dye. ***Blue circles*** indicate SSMs that are peptide loaded, as indicated by the loading marker, and are also positive for FM4-64. ***Green circles*** indicate SSMs that have been cryoloaded but fail to take up FM4-64. The ***red circle*** indicates an SSM that was not cryoloaded but was able to take up FM4-64. **(B)** Histogram of percent ± SE of dextran-positive SSMs that were FM4-64 positive from four experiments. Statistical test to the dextran-only control and HEADEDDWC, respectively, for each treatment are indicated as: *t*-test, ^∗^*p* < 0.05, ^∗∗^*p* < 0.01. Analysis by ANOVA (Bonferroni–Holm) confirmed a reduction in dye-uptake with HQARRVPNGY (*p* << 0.01).

We used cryoloading/styrlyl dye uptake (Nath et al., 2014) to test if the effect observed in the cell-free SV-PD assay predicts the effect of the peptide blockers on SV recycling in intact synaptosomes. Test and control peptides were cryoloaded into the SSMs together with an inert fluorescent dextran marker. The fraction of individual dextran-positive SSMs that recycled SVs was assayed by styryl dye uptake (**Figure 4A**), as described (Nath et al., 2014; Wong et al., 2014). A blinded protocol was used: the investigator carrying out the peptide loading and imaging was unaware of the treatments until after each experiment was completed and analyzed.

Styryl dye uptake was indistinguishable between the dextran-only controls and with the control peptide HEADEDDWC and also several of the test peptides, including ATNSGRSSRT, SYVSSLTSQS, and GTGTRGRSYY. A significant, but moderate reduction was observed with HYTLGLNTGP (*p* < 0.02). However, HQARRVPNGY caused a marked, ∼55% reduction (compared to untreated control) in dye-positive SSMs (*p* < 0.001; **Figure 4B**).

#### Peptide Combinations

We carried out a number of pilot experiments to test if SV-PD or styryl dye uptake could be further inhibited using various combination of the five mimetic peptides. However, since none of the combinations tested resulted in qualitatively greater effect than with the HQARRVPNGY peptide alone we presume that the primary SV binding site is within this 10 aa stretch.

#### Peptide Blockers and the Capture of the SV Accessory Protein RIM1/2

**FIGURE 5:**
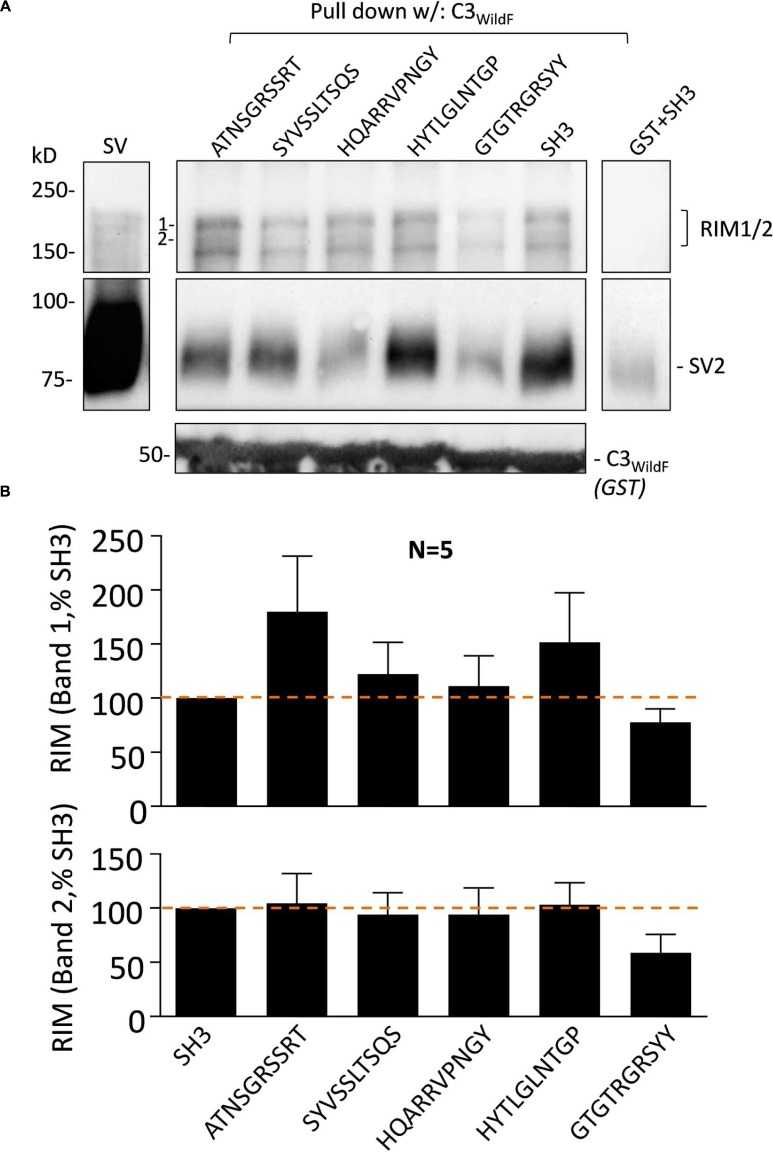
**Synaptic vesicle accessory protein RIM1/2 recovery is not blocked by HQARRVPNGY.** **(A)** As in **Figure 3**, purified SVs were treated with blocking or control peptides (0.6 mM each) followed by SV-PD with C3_WildF_. SH3 peptide was used as a control. As above, HQARRVPNGY markedly inhibited SV capture compared to controls as indicated in the SV2 immunoblot. GST + SH3 control lane shows protein capture in the absence of the C3-FLAG protein. **(B)** Mean + SE RIM1/2 higher (band #1, *upper histogram*) and lower (band #2, *lower histogram*) molecular weight band intensities were normalized to the SH3 control from the same experiment and plotted and were analyzed as in **Figure 2**. HQARRVPNGY had no significant effect on RIM1/2 capture as assessed by protein band intensity 1 or 2 (*as labeled*). Lower mean RIM1/2 intensities were observed with GTGTRGRSYY for both quantified bands but this failed to reach statistical significance (*t*-test, 0.1 > *p* > 0.05).

The effect of the peptide blockers and controls on SV-PD was generally consistent regardless of which SV integral membrane protein was used as the SV capture indicator. There has been considerable interest in the role of RIM1/2 in SV recruitment and hence, we also blotted for this protein in our SV-PD experiments. As reported previously (Wong and Stanley, 2010), RIM1/2 protein bands detected by Western blot of the SVs were typically not intense (**Figure 5A**). Interestingly, the mimetic peptide generated a different pattern of RIM1/2 recovery than for the integral SV proteins, as detailed above. Using C3_WildF_ as the bait protein, RIM1/2 was detected in the presence of all test or control peptides (**Figure 5A**). Densitometry analysis of two high-molecular weight bands in the RIM ladder (1 and 2 in **Figure 5A**) failed to demonstrate inhibition by HQARRVPNGY (**Figure 5B**). The mean intensity was lower in the presence of GTGTRGRSYY peptide but this failed to reach statistical significance (0.1 > *p* > 0.05). The C3_GST_ fusion protein (**Figure 1A**), that lacks the distal FLAG tag and is identical to the native protein gave very similar results (data not shown).

#### The HQARRVPNGY Region is Necessary for Effective SV Capture by the C3 Region

**FIGURE 6:**
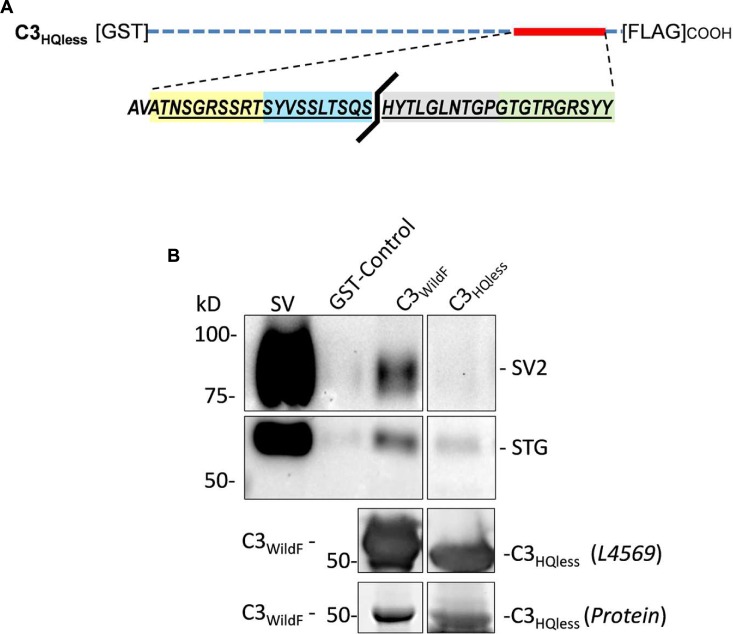
**Deletion of HQARRVPNGY from the C-terminal C3 region greatly reduces SV capture.** **(A)** A GST-tagged C3 fusion protein, C3_HQless_, was generated that was identical to C3_WildF_ but without the HQARRVPNGY sequence. **(B)** The molecular weight of the GST-C3_HQless_ band was confirmed by identification in a Western blot by both anti-GST (on the N terminal end, data not shown) and L4569, the antibody directed against the distal tip of the CaV2.2 C-terminal (beyond the deleted region). SV-PD was markedly reduced with C3_HQless_ compared to C3_WildF_. In the blot shown the concentration of C3_HQless_ was equal or higher than that of C3_WildF_, as demonstrated in a two-step method. First, we used L4569 to identify the protein band corresponding to each fusion protein. Since these bands were saturated in the blot, even with very short exposures, they could not be used for quantification. However, we also identified protein bands using a ‘Stain-free’ method and these bands were not saturated. Based on the L4569 probe we quantified the fusion protein bands by densitometry to obtain a C3_WildF_/C3_HQless_ ratio of 0.81. Thus, the SV2 bands were far darker with C3_WildF_ despite a lower concentration of the fusion protein. Similar results were observed in two additional experiments.

The peptide block experiments above identify the HQARRVPNGY region as containing the binding site for the SV onto the distal C-terminal. To rule out the possibility that the peptide might interfere with SV-PD by an anomalous mechanism we created a new GST fusion protein, C3_HQless_, that was identical to C3_WildF_ which lacks HQARRVPNGY (**Figure 6A**). Western blots of the C3_HQless_ fusion protein probed with FLAG (*not shown*) or anti-CaV2.2 C-terminal tip (L4569) antibody exhibited bands with a molecular weight that was, as expected, slightly lower than for C3_WildF_ (**Figure 6B**). SV-PD was markedly reduced (*N* = 3) using C3_HQless_ as the bait in comparison with C3_WildF_ (**Figure 6B**), providing independent support for the conclusion that HQARRVPNGY contains a key C3 region SV binding aa sequence. This region is the focus of the rest of this study though the residual SV-PD may indicate a secondary binding site. Further studies will be necessary to determine if there is any significant residual SV binding by the rest of the C3 region.

#### Identification of the HQARRVPNGY Binding Motif: Bioinformatics Analysis

**FIGURE 7:**
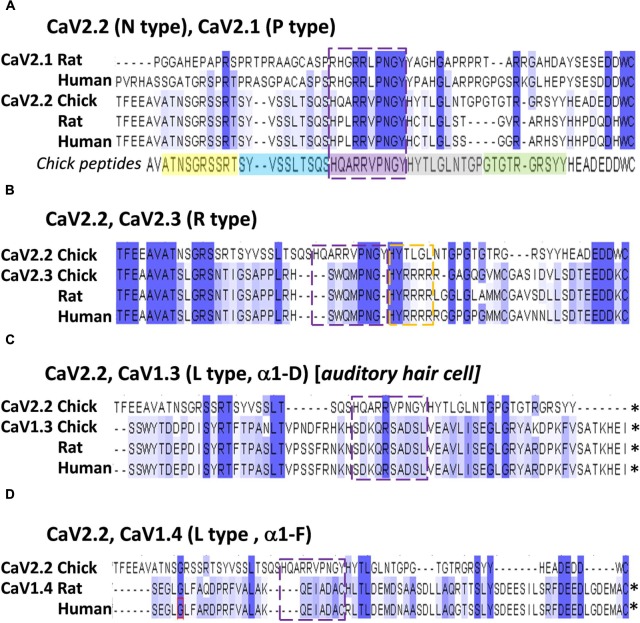
**The HQARRVPNGY peptide region is conserved across fast-transmission presynaptic calcium channel types.** Each panel shows an alignment of the chick CaV2.2 distal C3 region, as examined in this study, with the indicated CaV channel types from chick, rat, and human (*as available*). **(A)** CaV2.1 and CaV2.2. The five mimetic peptides (corresponding to chick CaV2.2) are indicated under the alignment. **(B)** CaV2.3. **(C)** CaV1.3. **(D)** CaV1.4. The HQARRVPNGY peptide sequence is indicated by the dashed box in each panel. ^∗^Distal aa extends beyond the diagram. Numbers above each set reflect the aligned protein set (including gaps), and do not correspond to any individual channel type.

Alignment of chick, rat and human CaV2.2 C-terminal sequences identified the conserved aa residues HxxRRVPNGY (**Figure 7A**), or HxxRRΦPNGY, where Φ is a hydrophobic residue. Since CaV2.1 channels are also important for transmitter release at fast transmitting terminals we extended the analysis to this channel type (note: chick CaV2.1 has not been cloned). This reduced the highly conserved region to RRΦPNGY, and served as our candidate binding motif.

#### Identification of the HQARRVPNGY Binding Motif: Blocking Peptide Analysis

**FIGURE 8:**
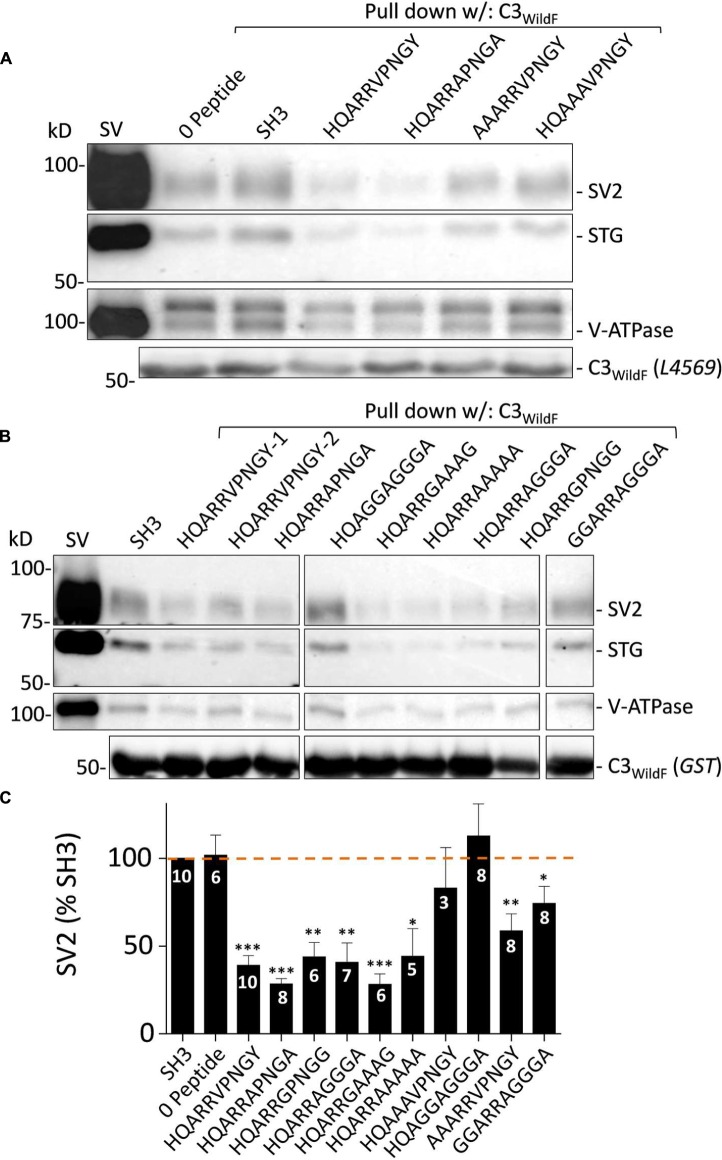
**Blocking peptide analysis of the SV binding motif.** **(A)** and **(B)** SV-PD assessed by immunoblot for SV integral membrane proteins in the presence of a range of HQARRVPNGY mutant peptides. SH3 and HQARRVPNGY served as negative and positive controls, respectively. **(C)**. SV2 bands densitometry histograms normalized to SH3. *N* = number of separate experiments. Values are means ± SE. Means were tested for significance from 100%: *t*-test, ^∗^*p* < 0.05, ^∗∗^*p* < 0.01, ^∗∗∗^*p* < 0.001. The data was also analyzed using by *post hoc* ANOVA, correcting for multiple comparisons (Bonferroni and Holm). The significance levels were similar: 0 peptide *p* > 0.05; HQARRVPNGY *p* < 0.01, HQARRAPNGA *p* < 0.01, HQARRGPNGG *p* < 0.05, AAARRVPNGY *p* > 0.05, GGARRAGGGA *p* > 0.05, HQARRAGGGA *p* < 0.05, HQARRGAAAG *p* < 0.01, HQARRAAAAA *p* < 0.05, HQAAAVPNGY *p* > 0.05, and HQAGGAGGGA *p* > 0.05. Note, however, that SV2 capture with AARRVPNGY and GGARRAGGGA peptides was reduced significantly with the *t*-test but not the ANOVA analysis.

To gain further insight into the SV binding site we created an additional series of blocking peptides in which amino acid residues of interest were mutated and tested with SV-PD (**Figures 8A–C**). SH3 or zero peptide, and HQARRVPNGY itself were used as negative and positive controls, respectively. Analysis focused on SV2 recovery but across the experiment STG recovery correlated strongly (*r* = 0.977, *p* < < 0.01)

Substitution of the two hydrophobic aa (Φ), V and Y, with A or G (HQARRAPNGA, HQARRGPNGG; **Figures 8A–C**) had no effect on block. Interestingly, and to our surprise, neither did substitution of the entire highly conserved ΦPNGY sequence HQARRAGGGA and HQARRGAAAG (**Figures 8B,C**). This result contradicted our bioinformatics-based hypothetical ΦPNGY-based SV-binding motif. We therefore turned our attention to the first five aa of HQARRVPNGY. Mutation of the arginine pair with either A or G (HQAAAVPNGY, HQAGGAGGGA; **Figures 8A–C**) eliminated inhibition of SV-PD, suggesting that these are essential. However, this aa pair was not sufficient for the full effect, because SV-PD inhibition was also reduced by replacement of the first two aa, HQ (AAARRVPNGY, GGARRAGGGA; **Figures 8A–C**). Without HQ the mean SV2 recovery was significantly less than 100% with both peptides, based on a *t*-test analysis but significance was not reached using ANOVA. Thus, our data is at least suggestive that the RR pair can cause a partial inhibition of SV-PD. In summary, our analysis identified the RR aa pair as essential for SV capture and this was enhanced by HQ.

## Discussion

The objective of this study was to identify an SV binding site within a 49 aa region of the distal C-terminal as reported earlier (Wong et al., 2014). We report first, that a 10 aa peptide, HQARRVPNGY, markedly inhibited SV capture using two different distal C3 region fusion proteins and also inhibited SV recycling, as assessed by depolarization-triggered styryl dye uptake. Within the HQARRVPNGY sequence we find that the HxxRR motif is required for effective SV capture by the channel C3 region.

Most of the experimental techniques and materials used in this study have been validated previously including fractionation, purification and characterization of SSMs and SVs (Juhaszova et al., 2000; Wong et al., 2013, 2014]; the SV-PD assay (Wong et al., 2013) and the peptide-cryoloading method combined with styryl dye assessment of SV recycling (Nath et al., 2014). Three fusion proteins were used. C3_WildF_ was reported previously (Wong et al., 2014) while C3_GST_, a new bait that lacks the terminal FLAG tag, is identical to the previously reported C3_Strep_ (Wong et al., 2014) but is tagged with GST to make it comparable with our other constructs. We also constructed C3_HQless_, which is identical to C3_WildF_ but with an excised HQARRVPNGY region.

In a previous study we reported that the distal tip peptide HEADEDDWC or its proximal segment, HEADE, did not inhibit SV-PD. We therefore assumed that we could use HEADE as a peptide control. To our surprise this peptide increased SV-PD, as confirmed by comparison to the SH3 peptide [that also did not inhibit SV-PD previously (Wong et al., 2014)] as well as all the inactive peptides used in this study (**Figures 2B**, **3B**, and **8**). This result might indicate that there is a region in the C-terminal that acts as an ‘on’-switch for binding. However, the failure to detect a similar enhancement with the overlapping, but longer HEADEDDWC peptide raises a question as to whether this has biological significance and will be explored in a future study.

Out of the five mimetic blocking peptides spanning the 49 aa binding site only HQARRVPNGY reliably and effectively inhibited SV-PD. A smaller effect was, however, also observed with GTGTRGRSYY. Of these two, only the former showed evidence of functional inhibition of SV recycling, supporting the hypothesis that this binding site has biological significance. We have not explored the role of GTGTRGRSYY further but the lack of functional inhibition with this peptide in the synaptosome cryoloading experiments combined with the marked reduction in SV-PD observed with C3_HQless_, which retains GTGTRGRSYY, suggests that its role is of less significance than that of the HQARRVPNGY region. A moderate reduction in styryl dye uptake was also observed with HYTLGLNTGP. Since this peptide had no detectable effect on SV-PD, we suppose that it interferes with an unrelated molecular interaction during SV recycling.

We used the SV-PD/peptide block strategy to carry out a systematic analysis of the binding site aa sequence (**Figure 8**). To our surprise, the ^∗^PNGY region could be mutated without any obvious loss of SV capture. However, removal of the arginine pair eliminated SV-PD. Interestingly, inhibition of SV-PD by the RR pair was markedly reduced, but not eliminated if HQ was also mutated. Since HQ could not capture SVs in the absence of the arginine pair we conclude that it plays a facilitatory role in SV binding.

**FIGURE 9:**
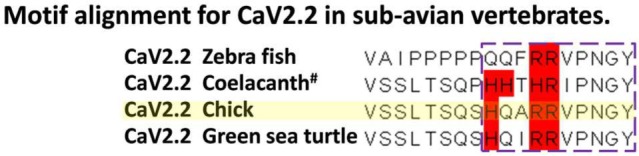
**Evolutionary conservation of the SV binding motif in early vertebrates.** Alignment of the region encompassing the chick CaV2.2 HQARR sequence for species from chick (yellow highlight) to fish. Positively charged aa are indicated in red. ^#^Sequence predicted by GenBank software but not cloned.

The HxxRR motif is conserved in CaV2.2 channels from chick to human, consistent with a significant biological role (**Figure 7A**) and the RR pair can be traced at least as far back as to fish (**Figure 9**). Interestingly, in the other principal release site-associated calcium channel, CaV2.1, the arginine pair is retained but the Hxx is replaced with an RHxRR (**Figure 7A**) in which the positive charge is not only retained but enhanced and may serve the same binding function.

Alignment of CaV2.3 channels, which can also support transmitter release at fast synapses, with CaV2.2 failed to identify any of the HxxRR elements (**Figure 7B**), raising a question as to how these channels may bind SVs. However, further scrutiny reveals a downstream H#RRRR(R) (**Figure 7B**) which might serve the same function. The more distantly related CaV1.3/CaV1.4, which gate transmitter release at sensory cell synapses (Vigh and Lasater, 2004; Brandt et al., 2005; Cho and von, 2012), such as at the higher vertebrate auditory hair cell, lack the HxxRR motif (**Figures 7C,D**). The motif is also absent in *Drosophila* CaV2.2-like channels (data not shown). Interestingly, both the sensory cell and *Drosophila* synapses exhibit a release site-associated structure, a ribbon in the former and T-bar in the latter, that is absent from fast transmitting synapses. Perhaps these structures usurp the SV binding site on the C-terminal. It is possible, therefore, that the SV binding site is specific for SV recycling at classical, fast transmitting release sites and that it evolved at an earlier stage during the diversification of CaV2 types (Anderson and Greenberg, 2001).

As expected, since they are all components of the same SVs, integral SV proteins were recovered in parallel in SV-PD experiments – thus, enhancement or inhibition of one such protein was mirrored by an equal enhancement or inhibition of its partner integral protein (Wong et al., 2014). However, immunoblot bands for the SV-associated protein RIM1/2 were faint and did not vary in parallel with the integral proteins (**Figure 5**). Thus, RIM1/2 capture was not reduced significantly by HQARRVPNGY nor was it enhanced by HEADE, supporting the hypothesis that RIM1/2 interacts with the channel C3 region by a mechanism that somehow differs from that with the integral SV proteins (Wong et al., 2014). A number of previous reports have failed to demonstrate RIM1/2 co-immunoprecipitation with the full-length CaV2.2 (Hibino et al., 2002; Khanna et al., 2006; Wong and Stanley, 2010), arguing against a common stable complex. However, co-variance of the channel and RIM1/2 at the intact transmitter release site does support the idea that the two proteins are components of separate interacting molecular complexes (Khanna et al., 2006). How RIM1/2 can be isolated with the purified SVs and yet fail to act in accord with the other SV proteins remains a mystery that will be the subject of further study.

This study does not address at which stage the C-terminal HxxRR contributes to SV recycling nor does it identify its putative SV binding partner. The length of the C-terminal and lack of predicted structure make it less attractive as a mechanism to bring the SV within range of the CaV calcium nanodomain (Wong et al., 2014; Stanley, 2015), at least without additional molecular partners. We have recently speculated that this binding region may play a role in delivering the SV to the release site, serving to capture the vesicle from the surrounding cytoplasm (Wong et al., 2014; Stanley, 2015) while additional and subsequent molecular interactions determine the precise molecular arrangement required for nanodomain-based release gating (Weber et al., 2010; Wong et al., 2014).

## Author Contributions

SG carried out the biochemistry experiments and their analysis; contributed to technical and conceptual innovation and experimental design. Contributed to statistical analysis and the writing of the manuscript. AN carried out and developed the functional (SV recycling) assays; commented on experimental design, commented on the manuscript. QL designed and created the constructs for the fusion proteins. Reviewed the manuscript. ES Conceived and funded project, supervised project and its analysis, reviewed data, wrote and was responsible for final edits of the manuscript and references. ES was also responsible for ethical and conflict issues.

## Conflict of Interest Statement

The authors declare that the research was conducted in the absence of any commercial or financial relationships that could be construed as a potential conflict of interest.

## Footnotes

1 http://statistica.mooo.com

## References

[B1] Anderson P. A. Greenberg R. M. (2001). Phylogeny of ion channels: clues to structure and function. *Comp. Biochem. Physiol. B Biochem. Mol. Biol.* 129 17–28. 10.1016/S1096-4959(01)00376-1 11337248

[B2] Brandt A. Khimich D. Moser T. (2005). Few CaV1.3 channels regulate the exocytosis of a synaptic vesicle at the hair cell ribbon synapse. *J. Neurosci.* 25 11577–11585. 10.1523/JNEUROSCI.3411-05.2005 16354915 PMC6726013

[B3] Cho S. von G. H. (2012). Ca(2+) influx and neurotransmitter release at ribbon synapses. *Cell Calcium* 52 208–216. 10.1016/j.ceca.2012.06.004 22776680 PMC3468149

[B4] Hibino H. Pironkova R. Onwumere O. Vologodskaia M. Hudspeth A. J. Lesage F. (2002). RIM binding proteins (RBPs) couple Rab3-interacting molecules (RIMs) to voltage-gated Ca(2+) channels. *Neuron* 34 411–423. 10.1016/S0896-6273(02)00667-0 11988172 PMC2151925

[B5] Juhaszova M. Church P. Blaustein M. P. Stanley E. F. (2000). Location of calcium transporters at presynaptic terminals. *Eur. J. Neurosci.* 12 839–846. 10.1046/j.1460-9568.2000.00974.x 10762313

[B6] Kaeser P. S. Deng L. Wang Y. Dulubova I. Liu X. Rizo J. (2011). RIM proteins tether Ca^2+^ channels to presynaptic active zones via a direct PDZ-domain interaction. *Cell* 144 282–295. 10.1016/j.cell.2010.12.029 21241895 PMC3063406

[B7] Khanna R. Li Q. Sun L. Collins T. J. Stanley E. F. (2006). N type Ca(2+) channels and RIM scaffold protein covary at the presynaptic transmitter release face but are components of independent protein complexes. *Neuroscience* 140 1201–1208. 10.1016/j.neuroscience.2006.04.053 16757118

[B8] Mochida S. Westenbroek R. E. Yokoyama C. T. Zhong H. Myers S. J. Scheuer T. (2003). Requirement for the synaptic protein interaction site for reconstitution of synaptic transmission by P/Q-type calcium channels. *Proc. Natl. Acad. Sci. U.S.A.* 100 2819–2824. 10.1073/pnas.262787699 12601156 PMC151424

[B9] Nath A. R. Chen R. H. Stanley E. F. (2014). Cryoloading: introducing large molecules into live synaptosomes. *Front. Cell. Neurosci.* 8:4 10.3389/fncel.2014.00004 PMC3899522 24478628

[B10] Seagar M. Leveque C. Charvin N. Marqueze B. Martin-Moutot N. Boudier J. A. (1999). Interactions between proteins implicated in exocytosis and voltage- gated calcium channels. *Philos. Trans. R. Soc. Lond. B Biol. Sci.* 354 289–297. 10.1098/rstb.1999.0380 10212477 PMC1692480

[B11] Sheng Z. H. Westenbroek R. E. Catterall W. A. (1998). Physical link and functional coupling of presynaptic calcium channels and the synaptic vesicle docking/fusion machinery. *J. Bioenerg. Biomembr.* 30 335–345. 10.1023/A:1021985521748 9758330

[B12] Stanley E. F. (1993). Single calcium channels and acetylcholine release at a presynaptic nerve terminal. *Neuron* 11 1007–1011. 10.1016/0896-6273(93)90214-C 8274272

[B13] Stanley E. F. (2015). Single calcium channel domain gating of synaptic vesicle fusion at fast synapses; analysis by graphic modeling. *Channels (Austin)* 9 324–333. 10.1080/19336950.2015.1098793 26457441 PMC4826128

[B14] Stanley E. F. (2016). The nanophysiology of fast transmitter release. *Trends Neurosci.* 39 183–197. 10.1016/j.tins.2016.01.005 26896416

[B15] Vigh J. Lasater E. M. (2004). L-type calcium channels mediate transmitter release in isolated, wide-field retinal amacrine cells. *Vis. Neurosci.* 21 129–134. 10.1017/S095252380404204X 15259564

[B16] Weber A. M. Wong F. K. Tufford A. R. Schlichter L. C. Matveev V. Stanley E. F. (2010). N-type Ca2+ channels carry the largest current: implications for nanodomains and transmitter release. *Nat. Neurosci.* 13 1348–1350. 10.1038/nn.2657 20953196

[B17] Wong F. K. Li Q. Stanley E. F. (2013). Synaptic vesicle capture by CaV2.2 calcium channels. *Front. Cell. Neurosci.* 7:101 10.3389/fncel.2013.00101 PMC3708276 23874268

[B18] Wong F. K. Nath A. R. Chen R. H. Gardezi S. R. Li Q. Stanley E. F. (2014). Synaptic vesicle tethering and the CaV2.2 distal C-terminal. *Front. Cell. Neurosci.* 8:71 10.3389/fncel.2014.00071 PMC3945931 24639630

[B19] Wong F. K. Stanley E. F. (2010). Rab3a interacting molecule (RIM) and the tethering of pre-synaptic transmitter release site-associated CaV2.2 calcium channels. *J. Neurochem.* 112 463–473. 10.1111/j.1471-4159.2009.06466.x 19878533

